# Phthalates, Bisphenol A, and Microbiological Investigations in Deep-Sea Shrimp *Aristaeomorpha foliacea* from Mediterranean Sea: Signs of Marine Anthropological Pollution

**DOI:** 10.3390/foods14213613

**Published:** 2025-10-23

**Authors:** Nicoletta De Vietro, Porzia Maiorano, Giovanna Mancini, Angela Carluccio, Giusy Diella, Antonella Francesca Savino, Valentina Spagnuolo, Francesco Triggiano, Roberto Carlucci, Giuseppe Strisciullo, Alessia Di Gilio, Jolanda Palmisani, Antonella Maria Aresta, Francesco Bagordo, Gianluigi De Gennaro, Osvalda De Giglio, Roberta Iatta, Michele Camero, Gianvito Lanave, Maria Mastrodonato, Ezio Ranieri, Giovanni Scillitani, Pasquale Stefanizzi, Silvio Tafuri, Carlo Zambonin, Gianfranco D’Onghia, Giuseppina Caggiano

**Affiliations:** 1 Department of Biosciences, Biotechnologies and Environment (D.B.B.A.), University of Bari “Aldo Moro”, via E. Orabona 4, 70125 Bari, Italy; porzia.maiorano@uniba.it (P.M.); angela.carluccio@uniba.it (A.C.); roberto.carlucci@uniba.it (R.C.); alessia.digilio@uniba.it (A.D.G.); jolanda.palmisani@uniba.it (J.P.); antonellamaria.aresta@uniba.it (A.M.A.); gianluigi.degennaro@uniba.it (G.D.G.); maria.mastrodonato@uniba.it (M.M.); ezio.ranieri@uniba.it (E.R.); giovanni.scillitani@uniba.it (G.S.); carlo.zambonin@uniba.it (C.Z.); gianfranco.donghia@uniba.it (G.D.); 2 Centro Interdipartimentale di Ricerca per l’Analisi e la Gestione del Rischio nelle Emergenze Sanitarie e Ambientali (C.I.R.S.A.), University of Bari “Aldo Moro”, Piazza G. Cesare 11, 70124 Bari, Italy; giuseppe.strisciullo@uniba.it (G.S.); francesco.bagordo@uniba.it (F.B.); osvalda.degiglio@uniba.it (O.D.G.); roberta.iatta@uniba.it (R.I.); michele.camero@uniba.it (M.C.); gianvito.lanave@uniba.it (G.L.); pasquale.stefanizzi@uniba.it (P.S.); silvio.tafuri@uniba.it (S.T.); giuseppina.caggiano@uniba.it (G.C.); 3 Interdisciplinary Department of Medicine, University of Bari “Aldo Moro”, Piazza G. Cesare 11, 70124 Bari, Italy; giusy.diella@uniba.it (G.D.); antonellasavino8@yahoo.it (A.F.S.); valentina.spagnuolo@uniba.it (V.S.); francesco.triggiano@uniba.it (F.T.); 4 Department of Pharmacy-Drug Sciences, University of Bari “Aldo Moro”, via E. Orabona 4, 70125 Bari, Italy; 5 Department of Veterinary Medicine, University of Bari “Aldo Moro”, S.P. Casamassima km. 3, 70010 Bari, Italy

**Keywords:** sea pollution, plastic waste, PAEs, BPA, shrimp, SPME, chromatographic techniques, bacteria, fungi

## Abstract

Sea pollution caused by anthropological activities represents a risk both for the organisms that inhabit it and for humans themselves. Great attention is paid to plastic waste because it takes decades to decompose and fragments into microscopic pieces that can be easily dispersed and ingested by marine fauna. Polymeric materials, in general, are rich in plasticizers (phthalates, PAEs; and bisphenol A, BPA), substances recognized as toxic both for aquatic organisms and for humans who could ingest them once contaminated marine organisms were to enter their diet. In this work, effective analytical protocols based on the use of solid phase microextraction (SPME) coupled with chromatography techniques were employed to evaluate the presence of PAEs and BPA in the extracted pulp of shrimps of the commercial species *Aristaemorpha foliacea* from four different fishing stations in the Mediterranean Sea. In addition to chemical analysis, a comprehensive microbiological characterization was carried out to assess microbiological risk due to shrimps’ consumption. This dual approach provides a more complete evaluation of the impact of human pollution on these crustaceans, revealing both chemical contamination and potential biological disruptions that could pose a danger to food safety.

## 1. Introduction

Worldwide, coastal, and offshore ecosystems are facing diverse environmental pressures from overexploitation, soil erosion, and urban and industrial waste discharges. Therefore, the consequences of pollution in the marine environment pose a risk to human health through contaminated seafood and affect marine ecosystems that provide valuable services.

Although quali-quantitative pollution by different contaminants may vary across regions, it is clear that plastic material represents a critical threat, especially because polymeric waste is voluntarily or involuntarily abandoned by humans across different ecosystems [1,2,3,4] and then widely dispersed by atmospheric agents (wind, rain, etc.), sewage or drainage systems, erosion, and rivers in coastal and offshore ecosystems, where it tends to accumulate on the seafloor [5]. Every year, approximately 275 million tons of plastic waste are produced worldwide; between 4.8 and 12.7 million tons are deliberately dragged or dumped into the sea. It is estimated that the planet’s municipal solid waste will double within 15 years, much of it in the form of single-use plastic items (e.g., bottles, bags, balloons, packaging, etc.), and it is forecasted to reach 53 million tons per year in 2030, for plastic alone [6]. As this waste takes decades to decompose, the European Union (EU) has classified plastic pollution as a global problem that is being addressed through regular monitoring programs and policy measures [7,8,9,10].

Sunlight and sea waves are the main agents that degrade plastic debris that reaches the marine environment, until they are reduced to micro- (size < 5 mm) or nanoscopic (size < 1 mm) fragments [11,12]. Microplastics (MPs) are particularly insidious in the marine environment, as they can be easily carried by water currents and wind even at great distances from their source, so much so that they have been found both in surface or deep waters as well as in ocean sediments and biota worldwide [11,12]. Microplastics have become ubiquitous in the marine environment, although benthic ecosystems are considered the largest sink for plastic and MP contamination, leading to multiple interactions with the biota present. Various potential effects have been detected in species with different trophic levels [13], as well as in those with high commercial value. Recent studies highlighted how some decapod crustaceans living on the seabed, which are valuable resources, are particularly exposed to MPs [14]. This triggers concern about potential economic impacts and the risks of dietary exposure, especially for coastal human communities.

Plastic fragments are ingested by all marine animals, including fish, dolphins, seals, turtles, and crustaceans (e.g., shrimp), organisms that live at variable depths and feed plants and small animals [7,15,16,17,18]. Although it is not yet clear how much of this plastic is ingested by marine organisms, it is true that in recent decades, plastic fragments, from 1 to 20, have been found among numerous fish species that proliferate in different habitats (e.g., deep sea, estuarine waters, etc.) [16,17,19,20], with a frequency between 2% and 100% of the fish analyzed [16,21].

The effects of microplastics on the organisms that ingest them are not yet fully understood, but the possible consequences include reduced sense of smell [22], gastrointestinal damage, and a false feeling of satiety that could even cause death from malnutrition [23]. Furthermore, once they enter the food chain, microplastics could accumulate and be ingested by top predators, including humans, with potential harmful effects on their health [24]. This risk exposure is principally connected to recognized toxic molecules, widely used by plastic producers as “plasticizers”, such as phthalic acid esters, better known as phthalates (PAEs), and bisphenol A (BPA).

PAEs are a synthetic group of molecules produced from ortho-phthalic acid and aliphatic/aryl alcohols, generally employed to improve the plasticity, strength, and flexibility of plastic materials (e.g., polyethylene, PE; polystyrene, PS; polyvinyl chloride, PVC, etc.), simultaneously reducing their fragility [25]. The use of BPA, on the other hand, is essential in the production of polycarbonate (PC), PVC, and epoxy resins that coat metal cans for food and beverages [26].

PAEs and BPA are toxic compounds, and their toxicity is mainly due to their ability to damage the endocrine system of humans, to cause carcinomas and dysplasia, and to adversely affect the reproductive system [27,28].

The dangers of PAEs and BPA have long attracted global attention, so much so that as early as 1977, the United States Environmental Protection Agency (USA EPA) listed six PAEs as priority pollutants, including dimethyl phthalate (DMP), diethyl phthalate (DEP), dibutyl phthalate (DBP), benzyl butyl phthalate (BBP), diethyl hexyl phthalate DEHP, and di-n-octyl phthalate (DnOP). In 2017, the Committee for Risk Assessment and the Committee for Socioeconomic Analysis proposed banning the use of DEHP, DnBP, diisobutyl phthalate (DiBP), and BBP in all plastic materials that could cause exposure to PAEs by contact or inhalation, such as textiles, mattresses, footwear, flooring, office supplies and equipment, etc. Commission regulation (EU) 2024/3190 of 19 December 2024 prohibited the use of BPA and other bisphenol derivatives with harmonized classification for specific hazardous properties in materials and articles intended for food contact, starting from January 2025, granting a transitional period of 18 months [29].

Crustaceans, furthermore, may serve as reservoirs for pathogenic bacteria, acquired both from the marine environment and during post-harvest handling. Specifically, the health and hygiene risks associated with the consumption of raw shrimp are influenced by multiple factors, including the quality of source water, environmental pollution, harvesting and transportation methods, storage conditions, and hygiene standards maintained throughout processing and distribution [30,31]. The increasing consumption of raw or minimally processed seafood has raised considerable public health concerns. In particular, the rising popularity of raw seafood products—such as shellfish, shrimps, and raw fish preparations like sushi, tartare, and carpaccio or shrimp-derived pulp that often is consumed without prior cooking—may contribute to foodborne illnesses due to potential contamination with pathogenic microorganisms [32].

Therefore, the assessment of microbial contamination in seafood products, such as shrimp, is of particular interest to improve food safety, maintain product quality, and ensure consumer health.

A crustacean of high commercial value, caught through trawl fishing in the Mediterranean, at depths between 400 and 800 m, is the giant red shrimp (*Aristaemorpha foliacea*) [33]. This species is also an important fishery resource in the central Mediterranean, between the southern Adriatic and the north-western Ionian Sea, where its biology, population dynamics, and the effects of environmental factors on its distribution and abundance have long been studied [32,34,35]. Recently, a significant increase in the abundance of giant red shrimp, correlated with the reduction in fishing effort, the increase in the sea-bottom temperature, and the presence of refuge areas scarcely accessible to fishing, has been observed in the north-western Ionian Sea [36].

Unfortunately, despite the importance of this species as seafood, apart from a study on heavy metal concentration in muscle and cephalothorax [37] and recent evidence on plastic ingestion by the giant red shrimp [38,39], there are no data on both its chemical and microbiological contamination. 

The aim of this study was to investigate the potential ingestion of plastic residues by *Aristaemorpha foliacea* shrimp from the Mediterranean Sea by determining the concentration of plasticizers (PAEs and BPA) in their pulp (edible part even raw) by solid phase microextraction (SPME) coupled with chromatography techniques.

A comprehensive microbiological analysis was also conducted to put in evidence the overall chemical and biological risk on human health, following ingestion of these crustaceans.

## 2. Materials and Methods

### 2.1. Sampling

A total of 80 individuals of the giant red shrimp *Aristaeomorpha foliacea* were collected during the Mediterranean Trawl Survey (MEDITS) program, included in the EU Data Collection Framework, carried out in the north-western Ionian Sea during the summer of 2024.

During this survey a standardized protocol, which includes gear characteristics, haul duration, and sampling procedures, following a depth-stratified random design, from 10 to 800 m in depth, was adopted [40].

**Figure 1:**
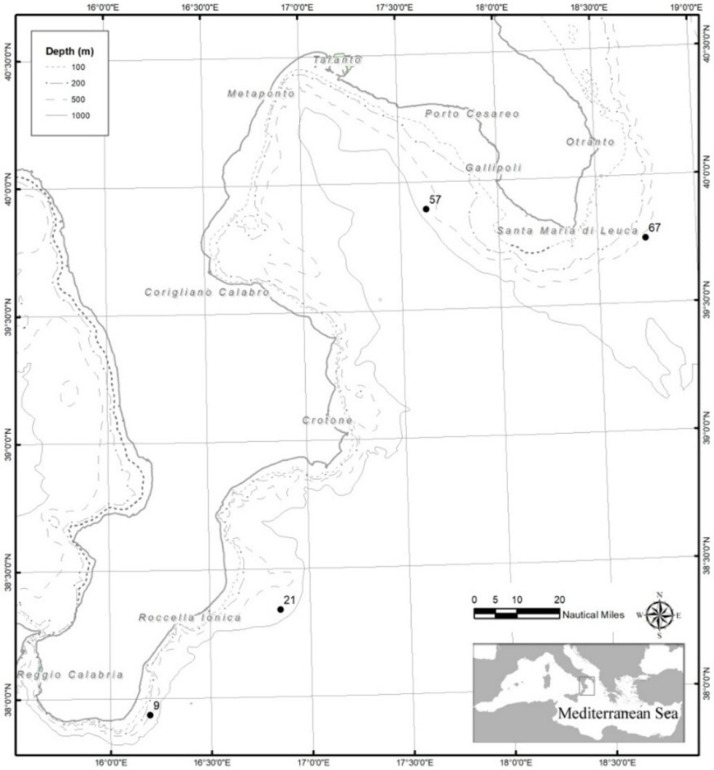
Map of the study areas in the north-western Ionian Sea, with indication of the sampling stations in the Apulian and southern Calabria.

The specimens of *Aristomorpha foliacea* were caught at different depths from four stations across two different areas of the north-western Ionian Sea, along the southern coast of Italy (Table 1; Figure 1). After collection, specimens were immediately frozen on board and stored at −20 °C until further processing and analysis.

### 2.2. Analytical Protocols for PAEs and BPA Determination

PAEs and BPA were quantified by applying effective analytical protocols based on the use of SPME coupled with gas chromatography/mass spectrometry (GC/MS) or high-performance liquid chromatography (HPLC), respectively, on shrimp-extracted pulp (edible part of the crustacean even without prior cooking).

#### 2.2.1. PAEs Determination

The selected target molecules were BBP, DBP, DEP, DMP, and DnOP. They are widely used in the production of plastic materials for food and non-food packaging, toys, etc., and are, as previously underlined, among the substances recognized as a priority by the USA EPA.

Standard compounds (purity > 99%) were purchased from *Sigma Aldrich* (St. Louis, MO, USA). Stock solutions, at a concentration level of 1 mg/mL, were prepared in sterile filtered ultrapure water (SFUW; *Sigma Aldrich*) with 20% (*w*/*v*) NaCl (*Sigma Aldrich*) and stored in glass vials at 8 °C [41]. Working solutions, daily obtained by diluting stock solutions with SFUW, were stored at 8 °C until use.

Following an experimental protocol previously successfully optimized for meat [42,43,44], 2 g of shrimp-extracted pulp sample was cut into small pieces, transferred into a centrifuge tube, and treated with 12 mL of an n-pentane/methanol (*Sigma Aldrich*) mixture in a 5:9 (*v*/*v*) ratio. The tube was shaken at room temperature on a vortex mixer for 5 min, followed by centrifugation for 10 min at 1150 RCF (SBS-LZ-4000/20, *Steinberg Systems*, Hamburg, Germany). The extraction process was repeated twice, and the supernatants were combined and dried by evaporation. The resulting residue was dissolved in 1.5 mL of a 20% NaCl aqueous solution and transferred into a 1.7 mL vial, before being subjected to SPME-GC/MS analysis.

A polydimethylsiloxane/divinylbenzene fiber (PDMS/DVB, diameter 65 µm, *Sigma Aldrich*) was used for the direct immersion SPME procedure (DI-SPME). PAE extraction was carried out under constant stirring for 20 min at 40 °C. At the end, the fiber was desorbed at 270 °C for 2 min in the GC injector, operated in splitless mode. To prevent a possible carryover effect, the fiber was kept at 200 °C for 30 min in the GC injector itself (90:10, split ratio) after the desorption step and before each subsequent extraction.

To preserve the robustness of the SPME fibers to more than 100 cycles, they were rinsed after each cycle and stored in fresh water overnight.

The GC/MS system used was a Finnigan TRACE GC ultra gas chromatograph (*Thermo Fisher Scientific*, Waltham, MA, USA) equipped with a split/splitless injector interfaced with an ion trap MS (Finnigan Polaris Q, *Thermo Fisher Scientific*). The capillary column used was a *Sigma Aldrich* SPB-5 fused silica (30 m length, 0.25 µm i.d., 0.25 µm film thickness) column, with helium (purity 99.9999%, *S.I.A.D.*, Bergamo, Italy) as the carrier gas, at a flow rate of 1 mL/min. The transfer line temperature was set at 260 °C. The oven temperature program was the same as that of Aresta et al. [44], modified as follows to reduce the elution times: initial temperature of 60 °C; ramp 10 °C/min from 60 °C to 260 °C; hold at 260 °C for 3 min. The mass spectrometer was operated in electron impact positive-ion mode (EI^+^), with the ion source temperature set at 250 °C. The electron energy was 70 eV, and the filament current was 150 µA.

Detection of individual PAEs was carried out by comparing the mass spectrum of each single analyte, obtained from a chromatogram acquired in TIC (total ion current; *m*/*z* range 50–450) mode of standard solutions (1 ng/mL) of each one of the five considered analytes, with the data contained in the NIST (National Institute of Standards & Technology) library implemented in the GC/MS system management software (Xcalibur). For all 5 PAEs, the relative retention times (RTs) and the characteristic ions, used for the subsequent acquisitions in SIM (selected ion monitoring) mode, were therefore identified and summarized in Table 2.

For the quantitative evaluation of PAEs in shrimp, the standard addition method (SAM) was used. Briefly, 100 µL of carefully prepared mixed standard solutions of PAEs at concentration levels in the range of 0.1–200 μg/mL were added to 2.0 g of the uncontaminated shrimp-extracted pulp sample, purchased from a local supplier and coming from the Atlantic Ocean. Each sample was then extracted and analyzed as described above, after 1 h of equilibration at room temperature.

The accuracy of the method was estimated through recovery studies assessed using the SAM. Low, medium, and large amounts of the five considered PAEs were added to the Atlantic Ocean shrimp-extracted pulp (reference sample), and extraction and analysis were performed as described previously. The mean recovery was calculated using the following formula: [47].

Recovery% = amount found/amount spiked × 100%(1)

Each measurement was repeated in triplicate.

Throughout all experimental procedures, the use of plastic objects of any kind (tips, containers, etc.) was always avoided, employing only glass and ceramic tools, preheated to 200 °C for 60 min before use. Blank analysis confirmed the absence of external contamination by PAEs.

#### 2.2.2. BPA Determination

BPA (purity ≥ 99%) was purchased from *Sigma-Aldrich*. HPLC-grade acetonitrile (purity ≥ 99.9%), hexane (purity ≥ 98%), and SFUW were also obtained from *Sigma-Aldrich*. Stock solutions of BPA were prepared in acetonitrile and stored in amber glass vials at 8 °C.

The analytical protocol used for shrimp analysis was developed by combining effective methodologies from previous studies [48,49,50].

Approximately 1 g of shrimp extracted pulp was homogenized in 2 mL of hexane, used as a defatting phase to remove lipids and reduce potential matrix interferences. Then, 2.5 mL of acetonitrile was added due to its intermediate polarity, which helps denature proteins and selectively solubilize BPA. The sample was vortexed for 5 min at room temperature, and the resulting suspension was centrifuged at 1150 RCF for 10 min (SBS-LZ-4000/20, *Steinberg Systems*). This led to the formation of two layers: an upper hexane-rich phase and a lower acetonitrile phase containing BPA. The acetonitrile fraction was carefully collected and evaporated to dryness; the residue was reconstituted in 1.5 mL of saline solution (7.5% *w*/*v* NaCl) and transferred into a 1.7 mL screw-cap vial with septum [48].

The aqueous solution was analyzed by SPME, using a polyacrylate (PA) fiber (85 µm thickness; *Sigma Aldrich*) selected for its high efficiency in extracting BPA from food matrices and simulants [48,49].

Before use, the fiber was conditioned for one hour in a vial containing the mobile phase; it was then immersed in the aqueous solution for 20 min at room temperature under constant magnetic stirring. After extraction, the fiber was desorbed for 5 min in a 200 µL microvial containing 150 µL of the mobile phase (static desorption mode). A 50 µL aliquot of the desorbed solution was injected into the HPLC system for BPA quantification.

To prevent potential carry-over phenomena, the fiber was immersed for 30 min in a vial containing the mobile phase, following desorption and prior to each subsequent extraction.

Chromatographic analyses were performed on a *Shimadzu* (Kyoto, Japan) Nexera system equipped with dual LC-30AD pumps, an RF-20AXS fluorescence detector (FLD), a SPD-M20A diode array detector (DAD), and an Accucore™ C18 analytical column (100 × 4.6 mm, 4 µm; *Thermo Scientific*). A matching guard column was employed. Data acquisition was managed using *LabService* software (v5.03).

HPLC analyses were carried out under isocratic conditions at room temperature [49], with a mobile phase consisting of acetonitrile and water (30:70, *v*/*v*) at a flow rate of 1 mL/min [51].

The identification of BPA was confirmed by comparing the signals obtained through fluorescence and UV detection. The FLD was set at an excitation wavelength of 280 nm and an emission wavelength of 310 nm, while UV spectra were acquired in the 220–500 nm range using a DAD. The peak observed at a retention time of 8.9 min in the fluorescence detector was attributed to BPA, based on the matching T_R_ in both detectors and the overlap of the UV absorption spectrum with that of the analytical standard, thereby confirming the identity of the analyte.

To ensure accuracy in BPA quantification, the SAM was employed. A calibration curve was built using FLD detection by adding BPA standard solutions, with concentrations ranging from 0.0008 to 0.08 mg/kg to 1 g of uncontaminated oceanic shrimp-extracted pulp sourced from a local supplier. These spiked samples were then processed and analyzed as previously described. As for PAEs, recoveries were determined using SAM by spiking Atlantic Ocean shrimp-extracted pulp (reference sample) with standard solutions of low, medium, and high concentration, employing Equation (1).

Each measurement was performed in triplicate.

Also in this case, the use of plastic objects during the experimental procedure was avoided, preferring, glass or ceramic tools preheated to 200 °C for 1 h. Blank analysis confirmed the absence of external contamination by BPA.

### 2.3. Microbiological Characterization

#### 2.3.1. Bacteria Investigation

The crustaceans, collected from the designed areas (Table 1), were transported under refrigeration (+4 °C) and processed within 24 h. According to the standardized methods detailed below, the pulp extracted from shrimps was examined for the following mesophilic microorganisms: total bacteria count (TBC), β-glucuronidase-positive *Escherichia coli*, Enterobacteriaceae, Enterococci, coagulase-positive staphylococci, *Listeria monocytogenes*, *Salmonella* spp., Shiga Toxin-Producing *E. coli* (STEC), and *Vibrio* spp.

For quantitative analysis, 10 g of each sample was added to 90 mL of Buffered Peptone Water (*Biolife Italiana srl*, Monza, Italy) and homogenized in a stomacher device (*Biosigma*, Cona (Venice), Italy), followed by serial dilution up to 10^−3^ prior to testing.

For the total bacteria count (TBC), 1 mL of each dilution was inoculated on Plate Count Agar (*Biolife Italiana srl*) by the pour plate method and incubated at 30 °C for 72 h (UNI EN ISO 4833-1:2013) [52].

For quantitative analysis of b-glucuronidase-positive *Escherichia coli*, each dilution was tested by the pour plate method on Tryptone Bile X-Glucuronide Agar (*Biolife Italiana srl*). The plates were incubated at 44 °C for 18–24 h. b-glucuronidase *E. coli*-positive colonies appear blue to blue-green after incubation (UNI ISO 16649-2:2010) [53].

The enumeration of coagulase-positive staphylococci was carried out by plating the samples on Baird Parker Agar (*Biolife Italiana srl*) and incubating at 37 °C for 48 h (ISO 6888-1:2018) [54]. Typical colonies, which appear black and gray, shiny and convex, and surrounded by a zone of clearing in the medium, were sub-cultured on Brain Heart Infusion Broth (*Biolife Italiana srl*), incubated at 30 °C for 24 h, and subsequently underwent coagulase testing with EDTA Plasma Coagulase (*Biolife Italiana srl*).

For the evaluation of Enterococci, samples were plated on Slanetz Bartley Agar (*Biolife Italiana srl*) at 36 ± 2 °C for 48 h. Presumptive colonies, with red, maroon, or pink colonies were subjected to biochemical and MALDI-TOF identification.

For the enumeration of Enterobacteriaceae, each dilution was tested by the pour plate method on Red bile Glucose (VRBG) Agar (*Microbiol s.r.l.*, Cagliari, Italy) and incubated at 37 °C for 24 h (ISO 21528-2: 2017) [55]. Presumptive pink or red-violet colonies with or without halos were subcultured on Dog Nutrient Agar (*Biolife Italiana srl*) for 24 h at 37 °C and subjected to confirmatory biochemical test.

For the evaluation of *Listeria monocytogenes*, 25 g of each sample was added to 225 mL of pre-enrichment Half-Fraser broth and incubated at 30 °C for 25 ± 1 h. Subsequently, 0.1 mL was inoculated in 10 mL of Fraser broth and incubated at 37 °C for 24 h. Each enrichment medium was plated on Ottaviani and Agosti Listeria Agar (ALOA; *Microbiol s.r.l.*) and Palcam Agar (*Biolife Italiana srl*) and incubated at 37 °C for 24 h, respectively. Presumptive colonies were subcultured on Columbia Blood Agar prior to confirmation tests (ISO 11290-1:2017) [56].

For the detection of *Salmonella* spp., 25 g of each sample was transferred to 225 mL of Buffered Peptone Water pre-enrichment (*Biolife Italiana srl*) and incubated at 37 °C for 24 h (ISO 6579-1: 2017) [57]. Subsequently, 0.1 mL of each sample was plated in three equidistant drops on Modified Semisolid Rappaport Vassiliadis (MSRV) Agar selective enrichment medium (*Microbiol s.r.l.*) and incubated at 41.5 °C for 24 h, and 1 mL was inoculated to 10 mL of Muller–Kauffmann Tetrathionate–Novobiocin Broth (MKTTn, *Biolife Italiana srl*) at 37 °C for 24 h. Then, the enrichment was plated on Xylose Lysine Desoxycholate Agar specific selective culture medium (*Biolife Italiana srl*) and Salmonella–Shigella agar (*Biomèrieux*, Marcy l’Etoile, France) and incubated at 37 °C for 24 h and 24–48 h, respectively. Presumptive colonies, with typical, black-centered colonies underwent further biochemical testing for identification.

The detection of Shiga Toxin-Producing *E. coli* (STEC) was performed by molecular methods: 25 g of shrimp pulp was transferred to 225 mL of prewarmed Buffered Peptone Water pre-enrichment (*Biolife Italiana srl*) and incubated at 41.5 ± 1 °C for 16–24 h. The DNA was extracted from the enrichment and tested by real-time polymerase chain reaction (PCR) PCR-iQ-Check STEC PCR Detection Kits (*BioRad*, Hercules, CA, USA) for the Shiga toxin genes (*stx1 and stx2*) and for the *eae* gene (intimin). At the same time, cultural investigation was performed by directly striking pre-enrichment on CHROMAgar^TM^ STEC (*CHROMagar^TM^*, Saint-Denise, France) and incubating at 35–37 °C for 18–24 h. No STEC was detected by either molecular or cultured methods.

For the evaluation of *Vibrio* spp., 25 g of each sample was homogenized in 225 mL of alkaline saline peptone water (ASPW) (*Microbiol s.r.l*.) the first enrichment and incubated at 41.5 °C for 6 ± 1 h (ISO 21872-1:2023) [58]. Subsequently 1 mL of the enrichment was inoculated into 10 mL of ASPW and incubated at 41.5 °C for 18 ± 1 h. Both enrichments were stroked on Thiosulfate Citrate Bile and Sucrose (TCBS) (*Biolife Italiana srl*) Agar plates and Soya peptone triphenyl tetrazolium chloride (TSTA) Agar (*HiMedia GmbH*, Einhausen, Germany) at 37 °C for 24 ± 3 h. Presumptive colonies were subjected to biochemical identification.

#### 2.3.2. Fungi Investigation

For the enumeration of fungi, 0,1 mL of each dilution was inoculated on Dichloran Rose Bengal Cloramphenicol (DRBC) Agar (*Liofilchem Srl*, Roseto degli Abruzzi, Termoli, Italy) and incubated at 25 °C for 5 days (ISO 21527:2018) [59].

## 3. Results and Discussion

### 3.1. PAEs and BPA

**Figure 2:**
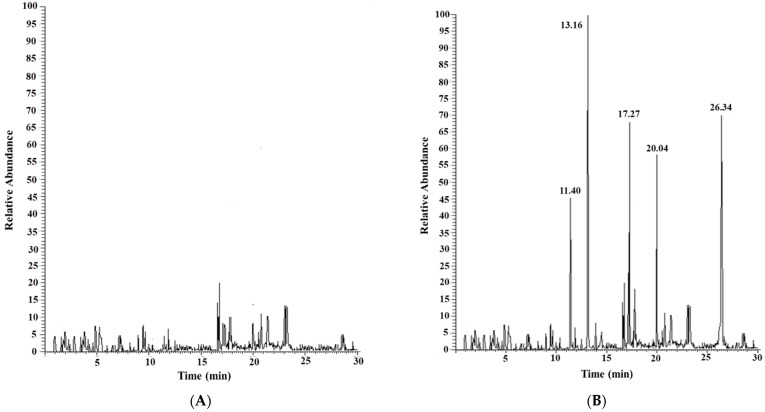
Chromatogram acquired in SIM of the extracted pulp sample (2 g) of Atlantic shrimp as purchased (**A**) and after the addition of 2 µg of each standard PAE (**B**).

Figure 2 shows a chromatogram acquired in SIM (acquisition parameters in Section 2.2.2) of the extracted pulp of shrimp (2 g) from the Atlantic Ocean, as purchased from a local supermarket (A), after the addition of 100 µL of a standard solution of the five selected PAEs at a concentration level of 20 µg/mL each (B).

No traces of the considered PAEs were found in the reference extracted shrimp pulp.

The analytical method was validated in terms of linearity, limits of detection (LODs) and quantification (LOQs), and precision. The results showed good linearity for all the analytes in the range of 0.005–1 mg/Kg, with correlation coefficients always better than 0.9924 (DEP). The LOD and LOQ, determined based on the signal-to-noise (S/N) ratio, ranged from 0.023 (DBP) to 0.037 (DEP) mg/Kg and from 0.076 (DBP) to 0.124 (DEP) mg/Kg, respectively. The intermediate precision of the method (expressed in % relative standard deviation, RSD%) for the same replicate sample ranged from 10.8 (DEP) to 24.5 (DnOP) and from 22.3 (DMP) to 34.3 (DBP), for within-day and between-day reliability, respectively. Table 3 summarizes all the analytical method validation parameters.

The accuracy of the method was estimated through recovery studies, conducted as described in Section 2.2.1.

For the five PAEs considered, the average recovery was in the range of 88.6 and 98.7% with a %RSD value less than 3.3. The SPME-GC/MS method applied, therefore, proved to be sufficiently precise, accurate, and sensitive for the simultaneous quantitative evaluation of the five compounds under study.

**Figure 3:**
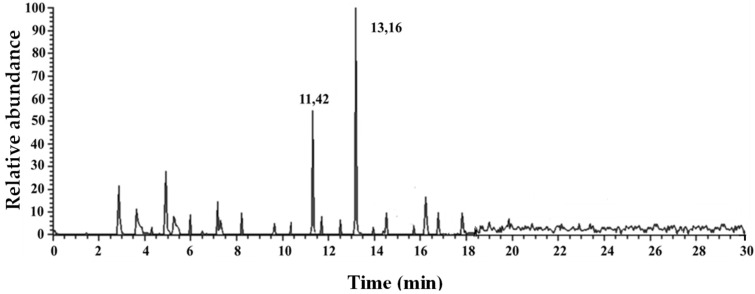
Chromatogram acquired from SIM of the extracted pulp (2 g) of shrimp from the Mediterranean Sea (St_9_Southern Calabria).

Figure 3 shows, as an example, the chromatogram acquired in SIM (acquisition parameters in Section 2.2.2) of the extracted pulp of shrimp (2 g) from the Mediterranean Sea (St_09_South Calabria).

Quantifiable levels of DBP, DEP, and DMP were found, as reported in Table 4, which shows the concentrations of the PAEs under examination found in the extracted pulp of shrimps from all four fishing stations across two different areas of the Mediterranean Sea.

**Figure 4:**
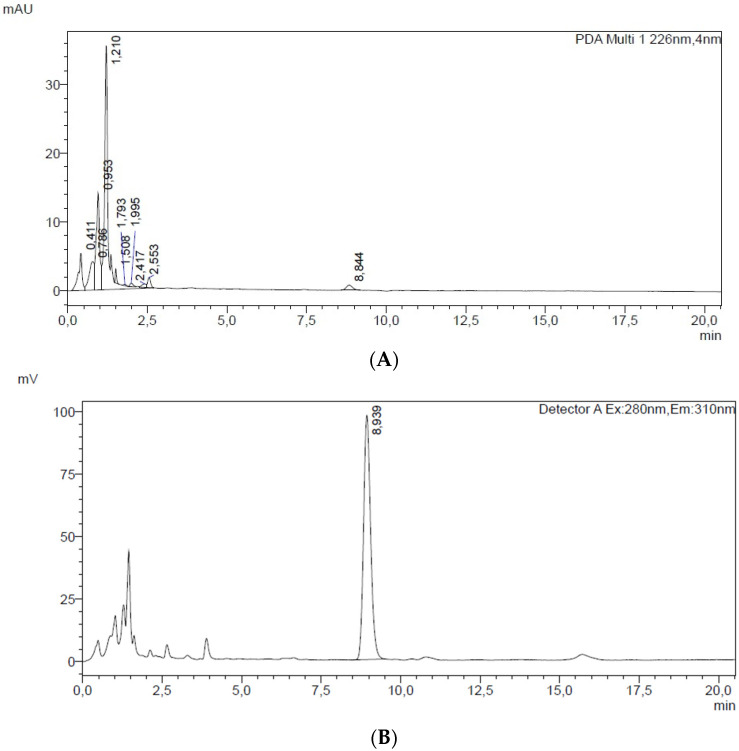
HPLC chromatograms with diode array (DAD) (**A**) and fluorescence (FLD) (**B**) detection of 1 g of shrimp extracted pulp after the addition of 1 µg of BPA standard.

The use of SPME coupled with HPLC-FLD detection is crucial for the analysis of non-volatile contaminants in shrimp-extracted pulp, as it offers pre-concentration and highly sensitive separation of low-concentration compounds. The fluorescence detector enhances the ability to detect specific contaminants, even at extremely low levels, due to its high selectivity and sensitivity. Figure 4 shows a HPLC chromatograms with diode array (DAD) (A) and fluorescence (FLD) (B) detection of shrimp-extracted pulp after the addition of BPA standard at a concentration level of 0.1 µg/g.

Appling the selected optimized experimental conditions (see Section 2.2.2), the instrument response (peak area) was proportional to the concentration, with a R^2^ value greater than 0.998. Method validation included assessment of linearity, the LOD and LOQ, and %RSD. The LOD and LOQ, determined based on the S/N ratio for PAEs, showed values of 0.0008 mg/kg for the LOD and 0.0042 mg/kg for the LOQ. Intra-day and inter-day precision values were 6.8 and 8.3 %RSD, respectively.

A recovery value of 98.8% with an %RSD of 2.4 permitted us to confirm that the method SPME-HPLC/FLD applied was precise, accurate, sensitive, and, consequently, useful for the quantitative evaluation of BPA.

Table 5 summarizes the BPA concentration levels in shrimp-extracted pulp samples from the selected four fishing stations across the Mediterranean Sea.

### 3.2. Microbiological

The total bacterial count showed significant variability between the samples, with values ranging from 920 to 84,000 CFU/g. The highest levels were observed at station 67, while the lowest count was recorded at station 21.

The fungal count was below the limit of quantification (<1 CFU/g) in all samples.

Similar results were observed for *E. coli* and Enterococci enumeration with, counts <1 CFU/g for all samples, except for stations 57 and 67, which showed 180 CFU/g and 50 CFU/g, respectively. Moreover, MALDI-TOF identification revealed the presence of *E. faecalis* for station 57 and 67.

The presence of Enterobacteriaceae was also generally very limited, with values below 1 CFU/g, except at station 9 (160 CFU/g), station 21 (80 CFU/g), and station 57 (220 CFU/g).

Coagulase-positive staphylococci were detected in all samples: station 9 (3000 CFU/g), station 21 (790 CFU/g), station 57 (1000 CFU/g) and station 67 (7300 CFU/g) (experimental results in Table 6).

None of the considered pathogens were detected in the sample analyzed (Table 7). However, *Proteus mirabilis*, which is a Gram-negative, rod-shaped, facultative anaerobic bacterium, was identified in the sample collected from station 57 using MALDI-TOF.

None of the pathogens were detected in the samples analyzed (see Table 7).

## 4. Discussion

All the crustaceans analyzed were contaminated by DEP and DMP at concentration levels between 0.15–0.27 mg/Kg and 0.13–0.17 mg/Kg, respectively. The shrimps from St_21 (Southern Calabria), moreover, contained 0.10 mg/Kg of DBP. Comparable levels of PAEs have also been found in shrimp from other parts of the world, e.g., Taiwanese aquafarms and major production areas in Taiwan (Yunlin, Chiayi, Tainan, Kaohsiung, and Pingtung) [60].

The presence of such quantities of plasticizers in the extracted pulp of the crustaceans analyzed can be traced back to ingestion, by the crustaceans themselves, of fragments of plastic materials dispersed in marine waters or deposited on the seabed. In the literature, in fact, there are studies that demonstrate the presence of plastics in the intestinal tracts of crustaceans caught in Italian seas. Bordbar Leila et al., for example, found that 14.6% of 621 giant red shrimp (*Aristaeomorpha foliacea*) of the eastern Ionian Sea contained, in their gut, plastic fibers with sizes in the range of 0.75–110.59 mm [61]. Moreover, the gastrointestinal tracts of shrimp belonging to the species under examination but originating from the Central Tyrrhenian Sea were examined by Laura Ciaralli et al., who demonstrated that, also in this case, 52% of the individuals analyzed had ingested microplastics [62].

DEP and DMP, recognized by recent studies as able to induce male and female reproductive toxicity, developmental damage, and liver effects [63,64], together with DnOP, are among the most detected persistent organic pollutants in the environment. These substances are not authorized to produce plastic materials and objects intended for contact with food [65], and so there is no specific migration limit (SML) for these compounds; therefore, the generic threshold limit of 60 mg/kg of food applies. According to Ministerial Decree 123 of 28 March 2003 [66], DEP may be present in quantities not exceeding 5% as the sum of all phthalates in plastic materials intended for contact with food.

As regards DBP, included by the EU list of substances that interfere with the endocrine system [46], as well as BBP, it is important to note that, according to EU Regulation 2023/1442 [67], the use of this substance is permitted to produce reusable plastic objects for contact with foods not containing fatty substances. Its SML is set at 0.3 mg/kg of food [65].

Analyzing our experimental results, it is possible to conclude that the concentration levels found for DBP, DEP, and DMP exceed the respective LOQ values and are always below the LMS permitted by law.

The BPA concentration level in the considered analyzed crustacean-extracted pulp ranged from 0.0008 (LOD level) to 0.0075 mg/kg. In most cases, the registered values exceeded the LOQ but were always significantly below the specific SML set by the EU [67], which is 0.05 mg/kg.

The detection of the considered residues of plasticizers in the extracted pulp of the shrimps analyzed confirms that plastic pollution of the seas undoubtedly also has a negative effect on the organisms that populate it since, evidently, small polymer fragments end up in the food they feed on and are not completely metabolized. Such contamination can therefore also represent a source of danger for human beings now, as the crustaceans considered enter our food chain. Among the various routes of exposure to BPA, in fact, the gastrointestinal one is predominant. Foods and beverages ingested through the diet represent the main source of contact with BPA for the global population [68], because they can contain significant quantities of BPA, generally released from the plastic containers or aluminum cans in which they are marketed. Specifically, Sakhi et al. [69] estimated that canned fish and fish products contained 1.2 μg/kg of BPA. Lorber et al. [70] found that the amount of BPA ingested daily due to the consumption of canned foods was equal to 0.0124 μg/kg of body weight, compared to a total daily amount of 0.0126 μg/kg of body weight, demonstrating that canned foods represent a significant portion of the daily BPA intake.

Once inside the human body, BPA acts as an endocrine disruptor, with estrogen- and antiandrogenic-like effects, causing damage to various tissues and organs, including the reproductive, immune, and neuroendocrine systems. Recently, it has been shown that BPA could induce carcinogenesis and mutagenesis in animal models [71].

Although the literature has documented an increase in foodborne pathogens (*Vibrio* spp., *Listeria monocytogenes*, and *Salmonella* spp.) in shrimp in recent years, the results of our study did not reveal the presence of these pathogens, including Shiga Toxin-Producing *Escherichia coli*.

Instead, high concentrations of *S. aureus* were detected, posing a risk to public health. *S. aureus* is recognized as one of the most significant foodborne pathogens worldwide [72].

It was shown by a study that was conducted in China in 2023 that, over time, contamination of shrimp with *S. aureus* has been increasing [72].

Some strains of *S. aureus* can produce thermostable enterotoxins that can maintain their biological and immune activity after treatment at 100 °C for 30 min [72].

Therefore, ingesting food contaminated with *S. aureus* can therefore cause various clinical symptoms, including gastroenteritis, vomiting, diarrhea, and abdominal cramps, typically within a few hours of exposure [73]. *S. aureus* characteristics, such as tolerance to high salt concentrations and low nutritional requirements, confer notable adaptability to aquatic environments, as well as to food processing and storage conditions [72]. Furthermore, it has been observed that *S. aureus* survives across a wide range of temperature (7 to 48.5 °C, optimal at 30–37 °C) [74]. Although several studies have reported that its occurrence may be attributable to the contamination of on-board freezing systems, inappropriate storage conditions, or inadequate hygienic practices during handling and processing [75,76], in our study, sampling was carried out under controlled conditions, using personal protection equipment (PPE) to avoid cross-contamination. Therefore, the presence of *S. aureus* in shrimp can be considered a case of pre-capture contamination originating from the marine environment. It is notable that this microorganism is not considered indigenous to seawater, but its presence in marine ecosystems is generally associated with anthropogenic activities, such as the inadequate wastewater discharge, surface runoff, and other land-based effluents [77].

*Enterococci*, known as part of the human and animal intestinal microflora, are widely used as fecal indicator bacteria of water. Their occurrence indicates possible fecal or environmental contamination; moreover, *Enterococcus* spp. represents a potential reservoir of antimicrobial resistance genes, with significant implications for food safety and public health [78].

Seafood can become contaminated naturally in the environment where it is usually harvested. Subsequently, shrimp are usually processed by washing and exported frozen. However, these processes are not sufficient to remove all pathogenic bacteria, meaning that they can reach the final consumer [79].

## 5. Conclusions

Marine pollution due to human activities, especially plastic waste that voluntarily or involuntary ends up in the sea, poses a serious risk both to the marine ecosystem and, indirectly, to human health. Marine organisms (e.g., fish, crustaceans, etc.) can ingest microscopic plastic residues, which then end up in our diet, as top predators at the end of the food chain.

Plastic contains plasticizers (e.g., PAEs and BPA), substances that, if ingested, can be particularly harmful to human health, recognized, for example, as endocrine disruptors, potentially responsible for carcinomas, dysplasia, and adverse effects on the reproductive system [27,28].

Plastic ingestion, with the occurrence of plastic fibers and MPs in the stomachs of *A. foliacea*, was recently documented in the Western and Eastern Mediterranean [39,61] but no data on the concentration of the toxic plasticizers in the edible muscle of this deep-sea shrimp have been reported so far.

In this study, effective analytical protocols based on the use of SPME coupled with chromatographic techniques were applied to determine PAE and BPA residues in the giant red shrimp *Aristaeomorpha foliacea*, across two different areas of the Mediterranean Sea. The results confirm and further extend the awareness of the high exposure of the deep-sea crustaceans to plastic pollution. All samples were found to be contaminated with DEP, DMP (in one case also by DBP), and BPA at concentration levels always below the maximum limits permitted by current regulations, suggesting a possible risk for human health related to their consumption. Furthermore, a comprehensive microbiological analysis completed the work and permitted us to evaluate the risks for human health associated with the ingestion of pathogens and fungi, if the examined crustaceans were consumed without prior cooking.

The consumption of seafood products, including crustaceans, is associated with multiple health benefits. Crustaceans are considered a source of high biological value proteins, omega-3 fatty acids, and antioxidants, which play a key role in reducing cardiovascular disease, with positive effects on lipid metabolism and cognitive function [80]. On the other hand, the consumption of crustaceans, especially raw, as is common in some gastronomic traditions, poses significant risks to human health. In fact, shrimps can act as reservoirs of pathogens, resulting in a risk of gastroenteritis and food poisoning. In terms of the One Health approach, which recognizes the interconnection between human, animal, and environmental health, ensuring the safety of seafood products requires reducing anthropogenic pollution and adopting sustainable practices aimed at protecting marine ecosystems. This integrated approach helps protect both public health and the integrity of the environment.

In conclusion, this dual approach provides a complete evaluation of the impact of human pollution on these crustaceans, revealing both chemical contamination and potential biological disruptions that could pose a danger to food safety.

## Figures and Tables

**Table 1:** Sampling station, with code, depth, and geographic coordinates of *Aristaeomorpha foliacea* specimens collected across two different areas of the north-western Ionian Sea (central Mediterranean Sea).

Area	Station Code	Depth (m)	Latitude	Longitude
Southern Calabria	9	543	3756163 N	01611752 E
	21	656	3820496 N	01651188 E
Apulia	57	592	3953622 N	01738057 E
	67	527	3945234 N	01844612 E

**Table 2:** RT and selected ions (*m*/*z*) for considered 5 PAEs.

*PAE*	*RT* (min)	*m*/*z*
BBP	20.04 ± 0.02	149, 206 [45]
DBP	17.27 ± 0.02	149, 205, 223 [42,44,45,46]
DEP	13.16 ± 0.02	105, 149, 177 [42,44,45,46]
DMP	11.40 ± 0.02	135, 163, 194 [42,45,46]
DnOP	26.34 ± 0.02	149, 279 [45]

**Table 3:** Analytical method validation parameters.

PAE	Slope	Correlation Coefficient (*R*^2^)	*LOD* (mg/Kg)	*LOQ* (mg/Kg)	Concentration Level (mg/Kg)					
					Within Day			Between Day		
					0.05	0.25	2.5	0.05	0.25	2.5
BBP	707,295	0.9940	0.030	0.100	22.1%	21.0%	19.7%	30.7%	28.8%	27.6%
DBP	501,948	0.9965	0.023	0.076	20.3%	21.6%	22.0%	33.8%	34.3%	30.2%
DEP	6 × 10^6^	0.9924	0.037	0.124	11.5%	10.8%	12.1%	22.4%	24.7%	25.3%
DMP	2 × 10^6^	0.9940	0.033	0.110	13.7%	12.5%	11.9%	23.5%	25.5%	22.3%
DnOP	607,682	0.9957	0.026	0.085	24.4%	23.1%	24.5%	30.2%	28.6%	29.4%

**Table 4:** Selected PAE concentration in shrimp-extracted pulp from 4 fishing stations across 2 different areas of the Mediterranean Sea.

Mediterranean Sea	BBP (mg/kg)	DBP (mg/kg)	DEP (mg/kg)	DMP (mg/kg)	DnOP (mg/kg)
ST_09_Southern Calabria	/	/	0.16 ± 0.03	0.13 ± 0.02	/
ST_21_Southern Calabria	/	0.10 ± 0.03	0.18 ± 0.03	0.17 ± 0.03	/
ST_57_Apulia	/	/	0.15 ± 0.03	0.16 ± 0.03	/
ST_67_Apulia	/	/	0.27 ± 0.05	0.17 ± 0.03	/

**Table 5:** Concentration levels of BPA in shrimps extracted pulp samples from 4 fishing stations across 2 different areas of the Mediterranean Sea.

MEDITERRANEAN SEA	BPA Concentration Level (mg/kg)
ST_09 (APULIA)	0.0058 ± 0.0003
ST_21_ (APULIA)	0.0075 ± 0.0005
ST_57 (SOUTHERN CALABRIA)	0.0058 ± 0.0003
ST_67 (SOUTHERN CALABRIA)	LOD level

**Table 6:** Microorganism counts obtained shrimps from the Mediterranean Sea.

Microorganism Count (CFU/g)						
Station	Total Bacteria Count	Total Fungi Count	*Escherichia coli*	*Enterobacteriaceae*	Positive-Coagulase *Staphylococci*	*Enterococci*
9	14,000	<1	<1	160	3000	<1
21	920	<1	<1	80	790	<1
57	1610	<1	<1	220	1000	180
67	84,000	<1	<1	<1	7300	50

**Table 7:** Detection of *Listeria monocytogenes*, *Salmonella* spp., and Shiga Toxin-Producing *Escherichia coli* (STEC), and *Vibrio* spp. in samples collected at four monitoring stations (9, 21, 57, 67).

Detection				
Station	*Listeria monocytogenes*	*Salmonella* spp.	Shiga Toxin-Producing *E. coli*	*Vibrio* spp.
9	Not detected	Not detected	Not detected	Not detected
21	Not detected	Not detected	Not detected	Not detected
57	Not detected	Not detected	Not detected	Not detected
67	Not detected	Not detected	Not detected	Not detected

## Data Availability

The original contributions presented in this study are included in the article. Further inquiries can be directed at the corresponding author.
